# Circular RNA CircPPP1CB Suppresses Tumorigenesis by Interacting With the MiR-1307-3p/SMG1 Axis in Human Bladder Cancer

**DOI:** 10.3389/fcell.2021.704683

**Published:** 2021-09-14

**Authors:** Feifan Wang, Yan Zhang, Xuejian Zhou, Xianwu Chen, Jiayong Xiang, Mengjing Fan, Yanlan Yu, Yueshu Cai, Hongshen Wu, Shihan Huang, Ning He, Zhenghui Hu, Guoqing Ding, Xiaodong Jin

**Affiliations:** ^1^Department of Urology, The First Affiliated Hospital, Zhejiang University School of Medicine, Hangzhou, China; ^2^Department of Pathology, Sir Run Run Shaw Hospital, Zhejiang University School of Medicine, Hangzhou, China; ^3^Department of Urology, Sir Run Run Shaw Hospital, Zhejiang University School of Medicine, Hangzhou, China

**Keywords:** circPPP1CB, bladder cancer, miR-1307-3p, SMG1, tumorigenesis

## Abstract

Circular RNA (circRNA) is a newly discovered endogenous non-coding RNA (ncRNA), which is characterized with a closed circular structure. A growing body of evidence has verified the vital roles of circRNAs in human cancer. In this research, we selected circPPP1CB as a study object by circRNA sequencing and quantitative real-time PCR (qRT-PCR) validation in human bladder cancer (BC). CircPPP1CB is downregulated in BC and is negatively correlated with clinical stages and histological grades. Functionally, circPPP1CB modulated cell growth, metastasis, and epithelial-to-mesenchymal transition (EMT) process *in vitro* and *in vivo*. Mechanically, we performed various experiments to verify the circPPP1CB/miR-1307-3p/SMG1 regulatory axis. Taken together, our results demonstrated that circPPP1CB participates in tumor growth, metastasis, and EMT process by interacting with the miR-1307-3p/SMG1 axis, and that circPPP1CB might be a novel therapeutic target and diagnostic biomarker in human BC.

## Introduction

Bladder cancer (BC), one of the most frequently occurring cancers worldwide, caused approximately 430,000 new-set cases in 2012 (Kamat et al., 2016), approximately 75% of which are non-muscle invasive (NMIBC) cases, with the rest diagnosed as muscle invasive (MIBC) or metastatic cases. Currently, morbidity and mortality rates of BC are still high due to limited diagnostic methods and clinical interventions (Antoni et al., 2017). Therefore, a further exploration of biomarker and treatment is sorely needed in BC.

Circular RNAs (circRNAs), a class of newly identified non-coding RNAs (ncRNAs), are generated *via* back-splicing (Guo et al., 2014; Kristensen et al., 2019). In the past decade, circRNA has no longer been considered as “junk” products. CircRNA circ-RanGAP1 modulates VEGFA to promote gastric cancer metastasis (Lu et al., 2020). CircRNA circAF4 acts as an oncogene *via* regulating MLL-AF4 fusion protein in MLL leukemia progression (Huang et al., 2019). Hsa_circRNA_101996 promotes cell proliferation and invasion in cervical cancer (Song et al., 2019). Besides, circRNAs could predict post-operative recurrence in colon cancer patients (Ju et al., 2019), and exosomal circRNAs are regarded as diagnostic biomarkers in non-small-cell lung cancer (Xian et al., 2020). Of note, in urological cancers, numerous studies explored the vital roles of circRNAs in modulating multiple biological functions (Luo et al., 2019; Mao et al., 2019; Xiong et al., 2019; Chen et al., 2020). Therefore, we focus on circRNAs as a potential biomarker or a therapeutic target in BC. Numerous studies have verified the vital effect of microRNAs (miRNAs) in the cell proliferation, apoptosis, and autophagy of BC (Braicu et al., 2019; Cai et al., 2019; Liang et al., 2019; Yan et al., 2019; Wang et al., 2020b). Interestingly, circRNAs could serve as sponges of miRNAs to modulate tumor biological behaviors in various human cancers (Sang et al., 2019; Song et al., 2019; Zheng et al., 2019; Chen et al., 2020; Wang et al., 2020a). Therefore, the regulatory network between circRNAs and miRNAs is worth investigating in BC.

SMG1 was involved in nonsense-mediated mRNA decay and was a member of PI3-kinase-like-related kinase family exerting vital roles in cellular stress and DNA damage responses (Yamashita et al., 2001; McIlwain et al., 2010; Chen et al., 2017; Ho et al., 2019). Loss of SMG1 completely leads to early embryonic lethal, but mice with SMG1 haploinsufficiency represent a predisposition to chronic inflammation, oxidative stress, and tumorigenesis (Roberts et al., 2013). Meanwhile, a growing body of evidence has confirmed that SMG1 acted as a tumor suppressor in various human cancers (Gubanova et al., 2013; Du et al., 2014; Han et al., 2014; Zhang et al., 2018; Mai et al., 2019; Li et al., 2020).

In the current research, circPPP1CB (hsa_circ_0119704), which was derived from *PPP1CB* gene, was identified and named. We found that circPPP1CB was decreased and acted as a tumor inhibitor in BC. Meanwhile, circPPP1CB was correlated with clinical stages and histological grades. Mechanically, circPPP1CB interacted with miR-1307-3p to modulate SMG1 expression and biological functions. Our results indicated that circPPP1CB may hold the potential of being a novel biomarker or a therapeutic target in human BC.

## Results

### CircPPP1CB (hsa_circ_0119704) Is Downregulated in Human Bladder Cancer Cells

**FIGURE 1:**
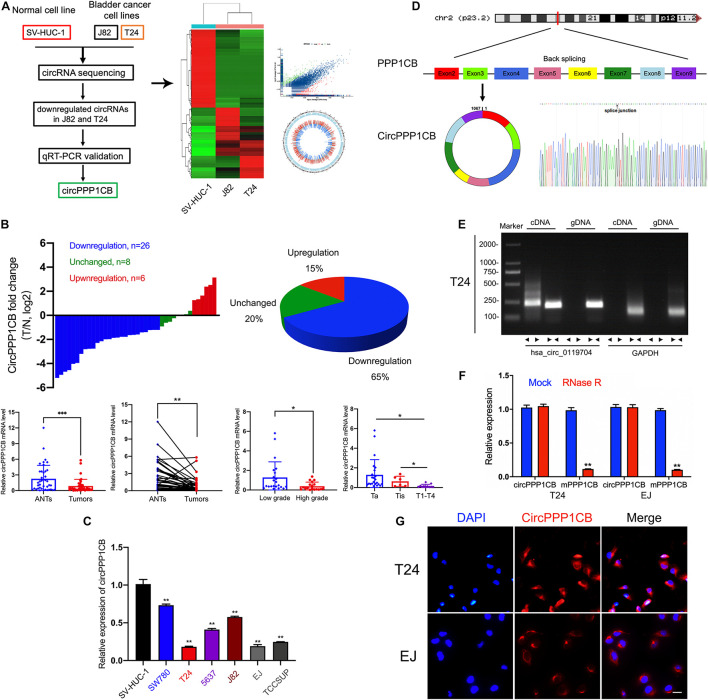
CircRNA sequencing and validation of circPPP1CB in bladder cancer tissues and cells. **(A)** CircRNA sequencing was performed in SV-HUC-1, J82, and T24 cell lines. A flow diagram, a heat map, and a volcano plot were presented. **(B)** CircPPP1CB levels were detected in surgical specimens collected from bladder cancer patients (*n* = 40). CircPPP1CB was downregulated in bladder cancer and was correlated with clinical stages and histological grades. **(C)**. Detection of circPPP1CB fold change in SV-HUC-1, SW780, T24, 5637, J82, EJ, and TCCSUP was performed by qRT-PCR. **(D)** CircPPP1CB was formed *via* circularization of exons 2, 3, 4, 5, 6, 7, 8, and 9 of PPP1CB. The head-to-tail splicing junction was also validated and indicated by Sanger sequencing. **(E)** CircPPP1CB could be amplified efficiently by divergent primers in cDNA in T24 cells. GAPDH was used as a negative control. **(F)** To verify the tolerance to RNase R, expression levels of circular and linear PPP1BC mRNAs were determined by qRT-PCR. **(G)** CircPPP1CB was predominantly located in the cytoplasm of bladder cancer cells. CircPPP1CB was stained by a specific probe (red), while nuclei were stained with DAPI (blue). Scale bars, 100 μm. Data are presented as mean ± SD of three independent experiments. *p < 0.05, **p < 0.01 vs. control group, ***p < 0.001.

We first performed circRNA sequencing between SV-HUC-1 (immortalized and normal bladder epithelial cell line) and cancer cells (T24 and J82) to explore the differentially expressed circRNAs (Figure 1A, GSE accession: GSE150142). According to the results of the following qPCR validation, circPPP1CB was chosen as a target circRNA in the current research. As shown in Figure 1B, circPPP1CB was downregulated remarkably in bladder tumor samples. The correlations between circPPP1CB and clinical and pathological features were also discovered in our cohort (*n* = 40). Meanwhile, the levels of circPPP1CB were also investigated in SV-HUC-1 cells and BC cells, and the results represented the same trend (Figure 1C). Next, according to the sequence in circBase, head-to-tail splicing was confirmed using Sanger sequencing with the RT-PCR product of circPPP1CB (Figure 1D). Since both trans-splicing and genomic rearrangements result in head-to-tail splicing (Jeck and Sharpless, 2014), divergent primers of circPPP1CB and convergent primers of PPP1CB mRNA were designed for PCR to eliminate the genomic rearrangements. The results of nucleic acid (cDNA and gDNA) electrophoresis experiment indicated that circPPP1CB could only be detected in cDNA but not gDNA in BC cells (Figure 1E). Another well-known characteristic of circRNA was its stable structure and resistance to digestion induced by RNase R (Chen, 2020). Therefore, we employed RNase R to verify the stability of circPPP1CB (Figure 1F). The results indicated that circPPP1CB could tolerate the elimination of RNase R, while the linear form PPP1CB was mostly digested by RNase R. In the end, RNA FISH assay showed that the cytoplasm was where circPPP1CB was localized in T24 and EJ (Figure 1G).

### Role of CircPPP1CB in Proliferation, Migration, and Invasion of T24 and EJ Cells

**FIGURE 2:**
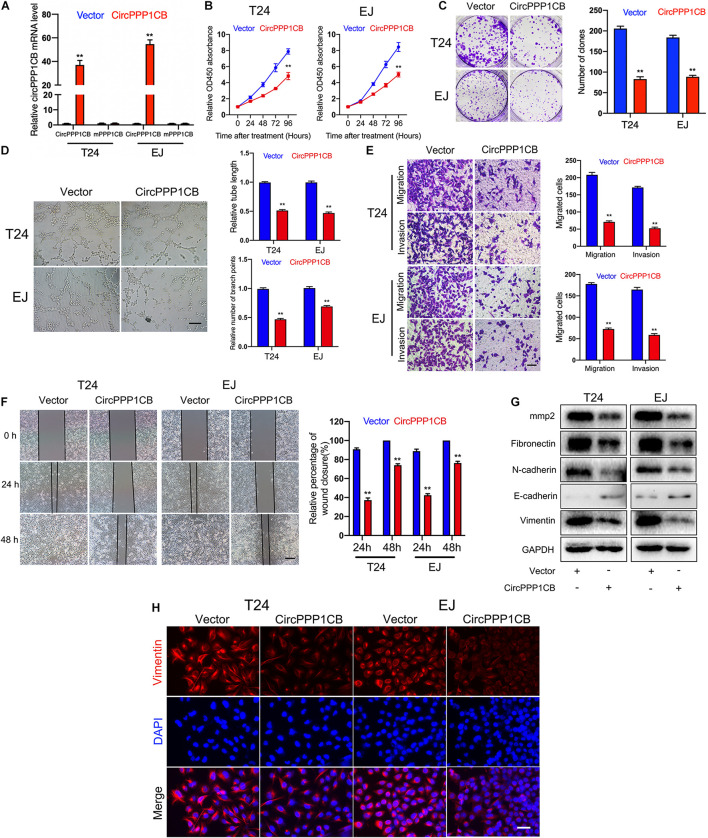
CircPPP1CB inhibits proliferation, angiogenesis, migration, and invasion of bladder cancer cells. **(A)** CircPPP1CB was significantly upregulated in circPPP1CB-overexpressing T24 and EJ cells. **(B)** CCK-8 assays indicated that CircPPP1CB reduced cell proliferation rate in bladder cancer cells. **(C)** Colony formation was evaluated by colony formation assay in negative control or circPPPC1B-overexpressing cells. **(D)** Tube formation assay was conducted in HUVECs with different conditional medium. Scale bars, 400 μm. **(E,F)** Transwell migration, invasion assay, and cell scratch assay indicated the biological effects of circPPP1CB in T24 and EJ cells. Scale bars, 200 and 400 μm, respectively. **(G)** EMT markers (fibronectin, N-cadherin, E-cadherin, and vimentin) and MMP2 were detected by western blotting in different T24 and EJ cells. **(H)** Immunofluorescence analysis of vimentin in circPPP1CB-overexpressing cells and negative control cells. Scale bars, 200 μm. **p < 0.01 vs. control group.

CircPPP1CB overexpressed T24 and EJ cells were constructed. qPCR showed the relative expressions of circPPP1CB and linear PPP1CB mRNA (Figure 2A). CCK-8 experiment suggested that circPPP1CB suppressed cell proliferation in BC (Figure 2B). Subsequent colony formation assay indicated the same trend (Figure 2C). Tube formation assay showed that circPPP1CB inhibited tumor angiogenesis in BC (Figure 2D). Meanwhile, cell scratch assay, transwell migration, and invasion assay further verified that the enhanced expression of circPPP1CB significantly suppressed migration and invasion in both cell lines (Figures 2E,F). According to the crucial role of epithelial-to-mesenchymal transition (EMT) in primary tumor initiation, progression, and metastasis (Bakir et al., 2020), we detected some EMT markers in circPPP1CB overexpressed cells. As presented in Figure 2G, circPPP1CB markedly reduced the levels of fibronectin, N-cadherin, and vimentin but enhanced E-cadherin expression. PPP1CB protein level was also detected (Supplementary Figure 4B). For further verification, we performed immunofluorescence by staining cells with vimentin antibody, and the results were similar with western blot (Figure 2H). Besides, we also confirmed a decreased level of matrix metalloproteases 2 (MMP2), a well-known protein related to cell adhesion and migration, in circPPP1CB overexpressed cells (Figure 2G). To clarify the biological functions of circPPP1CB more clearly, we knocked down circPPP1CB by transfecting siRNA, and the results were consistent with our previous experiments (Supplementary Figure 1). Taken together, the above results indicated a crucial role of circPPP1CB in various aspects of biological processes.

### CircPPP1CB Plays a Sponging Effect in Regulation of MiR-1307-3p in Bladder Cancer Cells

**FIGURE 3:**
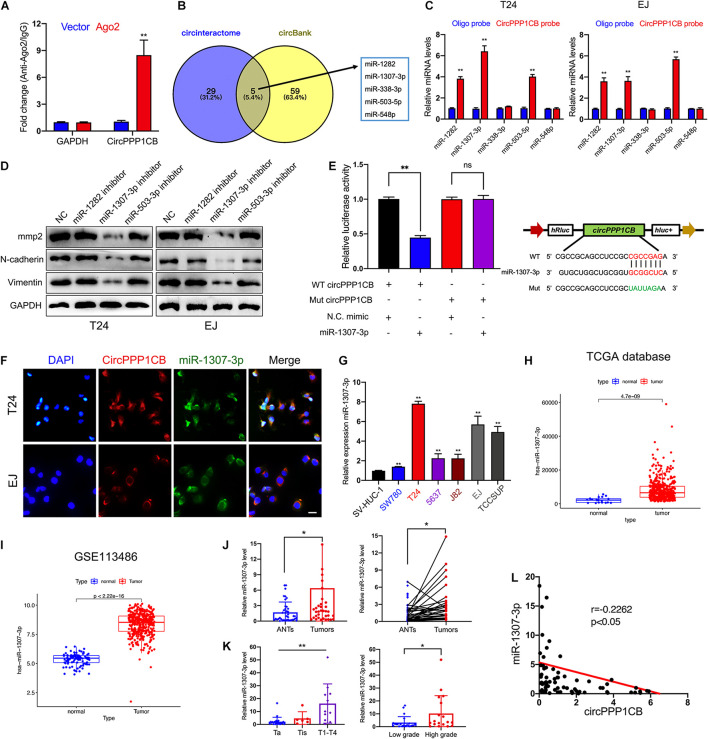
CircPPP1CB sponges miR-1307-3p in bladder cancer cells. **(A)** RNA immunoprecipitation for circPPP1CB in HEK-293 cells transfected with Ago2 plasmids. **(B)** Overlapping of target miRNAs predicted in circinteractome and circBank was illustrated by a Venn diagram. **(C)** A specific probe for circPPP1CB was used to capture five predicted miRNAs in RNA pull-down assay. MiR-1282, miR-1307-3p, and miR-503-5p were captured in both T24 and EJ cells. **(D)** Levels of EMT markers and MMP2 were assessed in cells transfected with miRNA inhibitors or negative control by western blotting. **(E)** Relative luciferase activities in HEK-293 cells transfected with luc-circPPP1CB or luc-circPPP1CB-mutant with or without miR-1307-3p mimics were determined. Wild and mutant sequences of circPPP1CB were presented. **(F)** RNA FISH indicated that circPPP1CB and miR-1307-3p were colocalized in the cytoplasm. Scale bar, 100 μm. **(G)** MiR-1307-3p levels were detected by qRT-PCR in SV-HUC-1 and six bladder cancer cell lines. **(H,I)** MiR-1307-3p was upregulated in bladder cancer in both TCGA database and GSE113486. **(J)** Dysregulation of miR-1307-3p in bladder cancer tissue compared with adjacent normal tissue was indicated in our own cohort (*n* = 40) by qRT-PCR. **(K)** MiR-1307-3p level was correlated with clinical stages and pathological grades. **(L)** Pearson analysis showed a negative correlation between miR-1307-3p and circPPP1CB in our cohort. *p < 0.05, **p < 0.01 vs. control group.

It is well-known that circRNAs could interact with miRNAs as competing endogenous RNAs (ceRNAs) and modulate their target genes and biological functions (Deng G. et al., 2020; Wang et al., 2020a; Zhan et al., 2020). Considering that the predominant localization of circPPP1CB was the cytoplasm, we supposed that circPPP1CB might exert its functions *via* sponging miRNAs. To verify our viewpoint, we performed RNA immunoprecipitation (RIP) using Ago2 antibody in control or Ago2 overexpressed 293T cells. The results indicated that more circPPP1CB was enriched by Ago2 antibody in Ago2 overexpressed cells compared with the control group (Figure 3A). We then predicted potential target miRNAs in circinteractome and circBank (Figure 3B). The overlapping miRNAs were caught by a biotinylated circPPP1CB probe and analyzed by quantitative real-time PCR (qRT-PCR) in an RNA pull-down assay. As shown in Figure 3C, miR-1282, miR-1307-3p, and miR-503-3p could be captured by circPPP1CB in both T24 and EJ cells. Next, we evaluated biological functions by detecting EMT markers and MMP2 in cells transfected with miRNA inhibitors. According to western blotting, only miR-1307-3p presented a remarkable influence on EMT process and cell adhesion (Figure 3D). Besides, these miRNAs had no effect on PPP1CB protein level (Supplementary Figure 4A). We subsequently constructed a mutant circPPP1CB luciferase reporter plasmid for verifying the predicted binding site. As shown in Figure 3E, miR-1307-3p mimic markedly suppressed the activity of luciferase reporter in wild-type but not mutated one. RNA FISH demonstrated the colocalization of circPPP1CB and miR-1307-3p in the cytoplasm of T24 and EJ cell lines (Figure 3F), supporting that circPPP1CB could interact with miR-1307-3p. Moreover, Figure 3G showed that was upregulated in bladder cancer cell lines. Besides, both our cohort, TCGA database, and GSE113486 implied upregulation of miR-1307-3p in tumor tissue, suggesting that miR-1307-3p might exert a vital effect in BC (Figures 3H–J). Besides, the expression level of miR-1307-3p was positively correlated with clinical stages and histological grades (Figure 3K). In the end, we also found a negative correlation between miR-1307-3p and circPPP1CB in our own BC patients (Figure 3L). Functionally, miR-1307-3p played a vital role in cell proliferation, tumor angiogenesis, migration, and invasion (Supplementary Figure 2). Therefore, we selected miR-1307-3p as the potential regulator in cricPPP1CB-mediated biological functions.

### CircPPP1CB Suppresses Tumor Growth, Metastasis, and EMT Process Through the CircPPP1CB/MiR-1307-3p Regulatory Pathway

**FIGURE 4:**
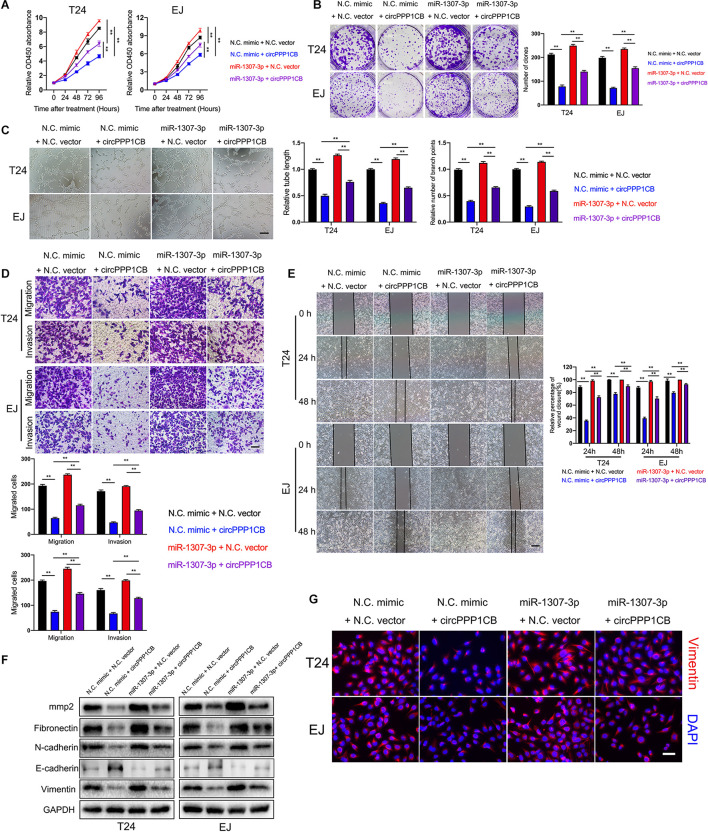
MiR-1307-3p reverses the biological effects induced by circPPP1CB. **(A,B)** Cell proliferation and colony formation ability were evaluated in cells under different conditions by CCK-8 assay and colony formation assay, respectively. **(C)** HUVECs were cultured with conditional media acquired from different bladder cancer cells to determine effects of miR-1307-3p and circPPP1CB on tumor angiogenesis. Scale bars: 400 μm. **(D–F)** The biological effects of circPPP1CB and miR-1307-3p were determined by transwell assay (scale bars: 200 μm), cell scratch assay (scale bars: 400 μm), and western blotting. **(G)** Expression levels of vimentin in cells were detected by immunofluorescence assay. Scale bars: 200 μm. **p < 0.01 vs. control group.

Functionally, rescue experiments were performed to confirm biological effects of the circPPP1CB/miR-1307-3p axis. CCK-8 and colony formation experiment indicated that circPPP1CB, together with miR-1307-3p, exerted lower rate of proliferation and colony formation than miR-1307-3p alone. Besides, miR-1307-3p rescued the biological functions impaired by circPPP1CB overexpression (Figures 4A,B), in consistent with the results of tube formation assay, cell scratch assay, transwell migration, and invasion assay (Figures 4C–E). To investigate EMT process and cell adhesion, we detected EMT markers and MMP2 by using specific antibodies. Western blotting showed that EMT process and cell adhesion were inhibited in circPPP1CB overexpressed cells but reversed by upregulation of miR-1307-3p. Meanwhile, miR-1307-3p-enhanced EMT process and cell adhesion could be partly attenuated by circPPP1CB (Figure 4F). The following immunofluorescence further confirmed the alternations of vimentin in different cells (Figure 4G). Taken together, we concluded that circPPP1CB suppressed miR-1307-3p-induced biological functions, forming the circPPP1CB/miR-1307-3p regulating pathway.

### SMG1 Is Targeted by MiR-1307-3p With Function as a Suppressor to Tumor

**FIGURE 5:**
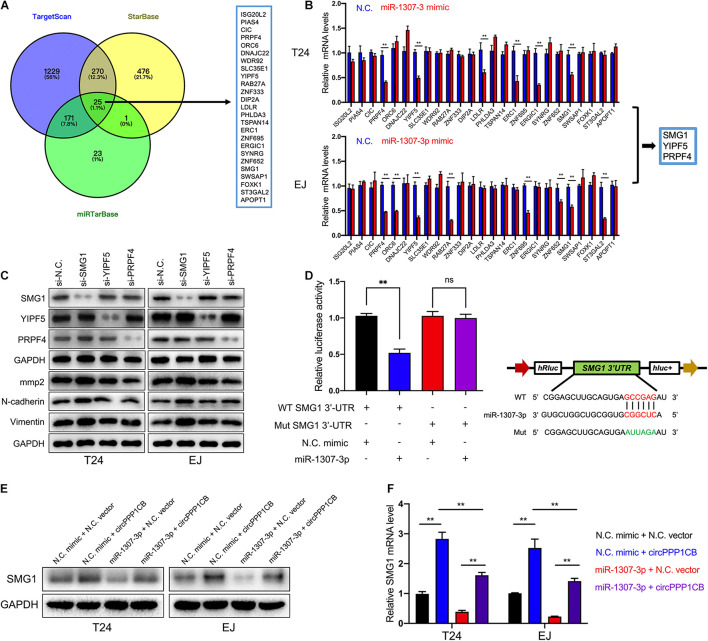
SMG1 is directly targeted by miR-1307-3p. **(A)** Overlapping of target genes of miR-1307-3p in TargetScan, Starbase, and miRTarBase. **(B)** Twenty-five potential genes were detected in control or circPPP1CB knockdown cells, and SMG1, WDR92, and PRPF4 were downregulated in both T24 and EJ. **(C)** Only SMG1 could induce the alternations of EMT markers and MMP2. **(D)** Luciferase reporter assay verified the interaction between SMG1 3’-UTR and miR-1307-3p. **(E,F)** A negative correlation between miR-1307-3p and circPPP1CB at mRNA level and protein level. *p < 0.05, **p < 0.01 vs. control group.

**FIGURE 6:**
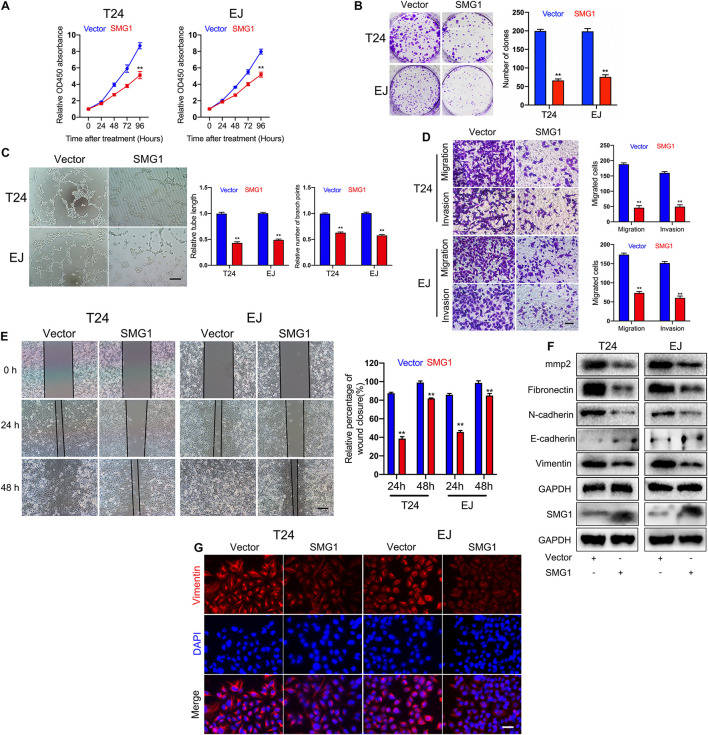
SMG1 serves as a tumor suppressor in bladder cancer cells. **(A)** SMG1 inhibited cell viability in T24 and EJ cells. **(B)** Colony formation assay was performed in bladder cancer cells transfected with vector or SMG1 plasmid. **(C)** Tube formation assay indicated that SMG1 was involved in tumor angiogenesis. Scale bars, 400 μm. **(D,E)** Transwell migration, invasion assay, and cell scratch assay showed the biological functions of SMG1. Scale bars, 200 and 400 μm, respectively. **(F)** Expression levels of SMG1, EMT-related genes, and MMP2 were determined by western blotting. **(G)** Immunofluorescence detection of vimentin in control and SMG1-overexpressing bladder cancer cells. Scale bars, 200 μm. *p < 0.05, **p < 0.01 vs. control group.

To unmask the deeper mechanism of the circPPP1CB/miR-1307-3p axis, we selected 25 potential genes through overlapping the bioinformatic predictions by TargetScan, Starbase, and miRTarBase (Figure 5A). As shown in Figure 5B, qRT-PCR indicated that SMG1, YIPF5, and PRPF4 were remarkably downregulated when circPPP1CB was inhibited. We then employed siRNAs to knock down these genes, and the results showed that only SMG1 served a key role in the EMT process (Figure 5C). Besides, luciferase reporter plasmids were constructed, containing different types (wild vs. mutant) of SMG1 3′-UTR sequence and transfected into 293T cells to verify the interaction (Figure 5D). Moreover, western blot and qRT-PCR showed that miR-1307-3p significantly decreased SMG1 at protein level and mRNA level, while circPPP1CB could upregulate SMG1 in both control and miR-1307-3p groups (Figures 5E,F). We further investigated the biological functions of SMG1 by overexpressing SMG1 in T24 and EJ. CCK-8 and colony formation experiment indicated that SMG1 restrained cell growth and proliferation (Figures 6A,B). SMG1 also suppressed tumor angiogenesis in human umbilical vein endothelial cells (HUVECs) (Figure 6C). Meanwhile, transwell assay and cell scratch assay showed that cell migrated and invasive abilities were impaired by SMG1 overexpression (Figures 6D,E). Western blotting showed the inhibition of EMT process and cell adhesion induced by SMG1 (Figure 6F). In the end, immunofluorescence confirmed the downregulation of vimentin in SMG1-overexpressing cells (Figure 6G). To further confirm the interaction between SMG1 and miR-1307-3p functionally, we overexpressed SMG1 or miR-1307-3p in T24 and EJ cells. The results verified the miR-1307-3p/SMG1 axis in human BC (Supplementary Figure 3).

### Overexpression of CircPPP1CB Inhibits Progression of Bladder Cancer *in vivo*

**FIGURE 7:**
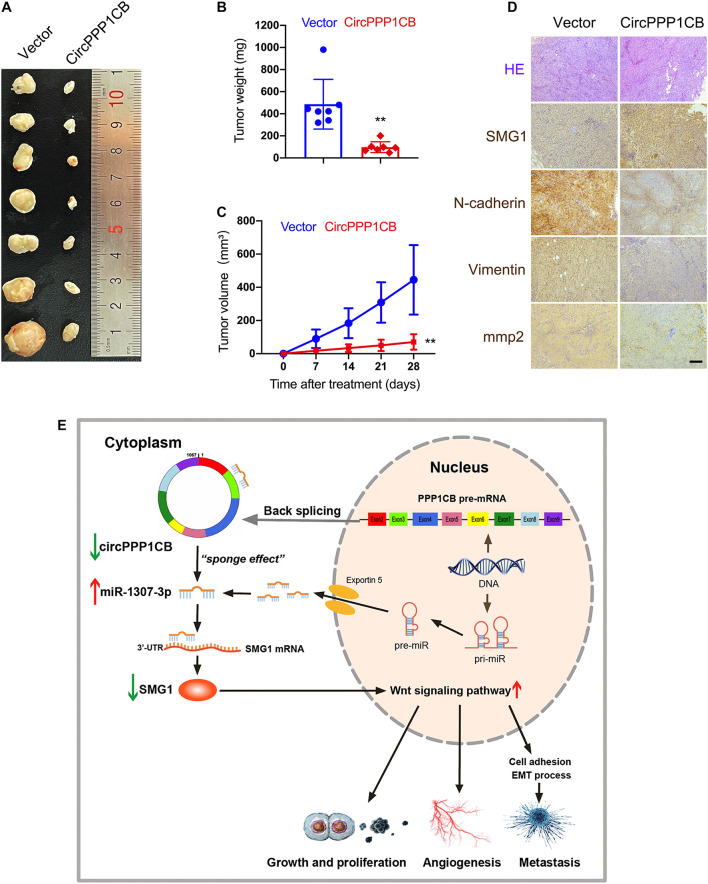
CircPPP1CB suppresses tumor growth *in vivo*. **(A)** An equal number of control or circPPP1CB-overexpressing T24 cells were injected subcutaneously to establish xenograft tumors. After 28 days, tumors were harvested (*n* = 7 each group). **(B,C)** Tumor weight was determined when mice were sacrificed, and tumor volumes were measured and calculated every week. **(D)** HE staining and immunohistochemistry (IHC) detection of SMG1, MMP2, and EMT markers in tumors. **(E)** A graphical abstract illustrating that circPPP1CB inhibits bladder cancer tumorigenesis by interacting with miR-1307-3p and regulating SMG1 expression. *p < 0.05, **p < 0.01 vs. control group.

CircPPP1CB-overexpressing and negative control T24 cells were subcutaneously injected into nude mice. Figures 7A–C indicate that overexpression of circPPP1CB reduced the weight and growing rate of tumor. Besides, alternations in MMP2 and EMT process biomarkers were determined by immunohistochemistry (IHC), and the results were in compliance with *in vitro* assays (Figure 7D). Moreover, SMG1 was remarkably upregulated in circPPP1CB-overexpressing xenografts (Figure 7D). In the end, to demonstrate our research in a more concisely and clear way, we presented a schematic illustration of the circPPP1CB/miR-1307-3p/SMG1 signaling pathway (Figure 7E). In summary, our current results demonstrated that circPPP1CB suppressed tumor growth *in vivo*.

## Discussion

Circular RNAs were first observed in eukaryotic cells approximately 40 years ago and were regarded as by-products or junk of abnormal splicing (Haddad and Lorenzen, 2019). However, as the studies of circRNAs move along, numerous circRNAs were verified to be dysregulated and modulated biological functions in human cancers, including colorectal cancer (Xu H. et al., 2020), cervical adenocarcinoma (Xu J. et al., 2020), hepatocellular carcinoma (Huang et al., 2020), prostate cancer (Deng Z.H. et al., 2020), and BC (Wang et al., 2020a). Meanwhile, roles of circRNAs in BC still remained unclear and were worthy of discovering. In the current study, we first identified a circRNA derived from *PPP1CB* gene and termed it circPPP1CB. Subsequent assays showed that CircPPP1CB was aberrantly lower and served as a tumor suppressor *via* the miR-1307-3p/SMG1 axis in human BC.

Different from the production and linear structure of mRNAs, circRNAs are formed through back-splicing of exons in protein-coding genes (Chen, 2020). Herein, circPPP1CB consisted of exons 2, 3, 5, 6, 7, 8, and 9 of *PPP1CB* gene and represented a circular structure *via* joint between the 3’ and 5’ sequences. Next, we employed RNase R to determine the stability of circPPP1CB. Besides, qRT-PCR detection indicated lower expression levels of circPPP1CB in both BC cells and tissues. Meanwhile, the correlation between circPPP1CB and clinical features was also unmasked. For more credible results, we would continue to collect clinical specimens and determine the expression levels of circPPP1CB in the future. Functionally, we verified the vital roles of circPPP1CB in cell proliferation, migration, invasion, and tumor angiogenesis of BC by overexpressing and inhibiting circPPP1CB in BC cells.

Considering the dysregulation and biological effects of circPPP1CB, it is reasonable to regard circPPP1CB as a possible diagnostic biomarker or a potential therapeutic target for BC. In the past few years, an increasing number of studies have proven the existence of circRNAs in body fluids, which indicated that circRNAs might be suitable to be diagnostic and prognostic biomarkers in human diseases. A recent study determined a circRNA profiling in plasma and explored its potential in diagnosis and prognosis of esophageal squamous cell cancer (Fan et al., 2019). Besides, a research established a landscape of circRNAs in human saliva by using high-throughput RNA sequencing (Bahn et al., 2015). Of note, Chen et al. (2018) verified the correlation between tumor metastasis and circPRMT5 acquired from serum and urine exosomes of BC patients. Our future study will collect different kinds of clinical specimens including urine, plasma, and tumor tissue and try to set up a database of circPPP1CB based on more BC patients. Moreover, to enhance the efficiency of circPPP1CB in clinical diagnosis and prognosis, other existed non-ncRNAs and biomedical index will be analyzed in this diagnostic and prognostic system. Numerous studies reported that circRNAs were involved in cancer progression and metastasis (Chen Y. et al., 2019; Hong et al., 2019; Xu et al., 2019), which indicated a promising therapeutic role of circPPP1CB. A recent article demonstrated that circRNA SCMH1 could be delivered *via* the extracellular vesicle to alleviate ischemic stroke in rodent and non-human primate models (Yang et al., 2020). Besides, circRNA 0001073 reduced tumor growth *in vivo* through nanoparticle delivery system in human breast cancer (Yi et al., 2020). Since circPPP1CB acted as a tumor suppressor in human BC, it is meaningful to explore safe and efficient methods to deliver exogenous circPPP1CB into BC cells *in vivo* in the future.

Circular RNAs are widely known as biological sponges of miRNAs to modulate varieties of downstream target genes and functions (Kristensen et al., 2019). Our previous study elucidated a circRIMS1/miR-433-3p/CCAR1 modulatory pathway in BC (Wang et al., 2020a). Besides, circRHOBTB3 could act as a sponge of miR-654-3p to suppress gastric cancer growth (Deng G. et al., 2020). Chen L.Y. et al. (2019) confirmed that circNSD2 served as a tumor promoter in colorectal cancer *via* targeting miR-199b-5p. In this research, circPPP1CB and miR-1307-3p were colocalized in the cytoplasm. Further experiments, such as RNA pull-down and luciferase reporter assay, confirmed the above sponge effect. Moreover, miR-1307-3p was confirmed to be a tumor promoter in BC. Functionally, circPPP1CB could reverse the biological effects of miR-1307-3p, and miR-1307-3p attenuated the suppressions of progressive characteristics induced by circPPP1CB overexpression. Mechanically, the circPPP1CB/miR-1307-3p axis directly targeted SMG1 to modulate biological functions. SMG1, a member of the phosphoinositide 3-kinase-related kinases family, acted as a novel tumor suppressor in various human cancers (Du et al., 2014; Mai et al., 2019; Yin et al., 2019; Li et al., 2020). In our study, we confirmed that SMG1 remarkably suppressed cell proliferation, migration, invasion, and tumor angiogenesis of BC. SMG1 was reported to exert its roles *via* activating the Wnt pathway in gastric cancer (Zhang et al., 2018). Since the Wnt signaling pathway was closely related to cell migration (Pai et al., 2017) and angiogenesis (Vallée et al., 2018a,b), according to these results, we supposed that the circPPP1CB/miR-1307-3p/SMG1 axis might regulate biological functions *via* modulating the Wnt pathway. However, the exact molecular mechanism underneath this process still needs further investigation.

In summary, circPPP1CB was dysregulated in BC and inhibited bladder cell growth, angiogenesis, migration, and EMT process *via* modulating the miR-1307-3p/SMG1 axis. Our results showed a novel perspective of clinical intervention in BC.

## Materials and Methods

### Ethical Approval

All experiments on animals acquired consent from the Ethics Committee of the First Affiliated Hospital, Zhejiang University School of Medicine. All programs were implemented strictly following the NIH Laboratory Guidelines for animals.

### Clinical Samples

Bladder tumor samples and adjacent control normal tissues were collected from the surgical specimens, which was approved by the Ethics Committee of the First Affiliated Hospital of Zhejiang University School of Medicine. Patients involved in this program all signed a written informed consent and agreed to this research.

### Cell Lines and Culture

HEK-293 cells, SV-HUC-1 cells (human immortalized bladder epithelium cell line), and human bladder carcinoma cells, including J82, SW780, T24, 5637, EJ, and TCCSUP cell lines, were purchased from the American Type Culture Collection (ATCC, United States). Dulbecco’s modified Eagle’s medium was used for HEK-293 cells culture. RPMI-1640 medium with 10% FBS (Gibco, United States) and 1% double antibiotics (penicillin/streptomycin) was used for culture of SV-HUC-1 and bladder carcinoma cell lines. All cell culture was implemented at 37°C in 5% CO_2_.

### RNA Extraction, Reverse Transcription, and qRT-PCR Analysis

TRIzol Reagent (Invitrogen, United States) was used to extract cell total RNA. When RNase R degradation experiment was conducted, total RNA (2 mg) was incubated in the presence or absence of 3 U/mg RNase R (Epicentre, United States) for 15 min at 37°C. Then, reverse transcription was conducted 1 μg total RNA using PrimeScript RT reagent kit (Takara Bio Inc., China). Subsequent detection of levels of circRNA and mRNA was performed with TB Green Premix Ex Taq II (Takara Bio Inc., China) kit on a QUANT5 PCR system (Applied Biosystems by Life Technologies, United States). GAPDH was regarded as an endogenous control. Besides, the All-in-One miRNA qRT-PCR detection kit (GeneCopoeia, United States) was used for miRNA analyses, and human U6 was taken as an endogenous control. Results were calculated using the 2^–ΔΔCt^ method. All the primers are presented in Supplementary Table 1.

### SiRNA and Mimics of MiRNAs

SiRNA and miRNA mimics were synthesized by RiboBio (Guangzhou, China).

### Plasmid and Lentivirus Construction

To construct circPPP1CB overexpression plasmids, cDNA containing circPPP1CB was created and embed into the pcD-ciR vector by GeneSeed (Guangzhou, China). Cells were transfected with pcD-ciR vector (containing a front circular frame and a back circular frame) using Lipofectamine 3000 (Invitrogen). Selection was conducted using puromycin for at least 6 days after lentivirus infection.

### Fluorescence *in situ* Hybridization

Probes of Cy3-circPPP1CB and FITC-miR-1307-3p were synthesized. Fluorescence *in situ* hybridization (FISH) detection kit (RiboBio, China) was used for hybridization experiments. All images were photographed with a Zeiss Axio Observer A1 (Carl Zeiss, Germany).

### Nucleic Acid Electrophoresis

Two percent agarose gel electrophoresis and TAE running buffer were used for the PCR products verification (120 V, 30 min). DNA marker was created using NormalRun 250 bp-II DNA ladder (Generay, Shanghai, China). Bands were observed and photographed under UV irradiation. Primer sequences are provided in Supplementary Table 1.

### Target Prediction of CircPPP1CB and MiR-1307-3p

We predicted potential miRNA-binding sites of circPPP1CB using the bioinformatics databases circinteractome^1^ and circBank.^2^ We used Starbase (Yang et al., 2011), miRTarBase, and TargetScan^3^ to predict target genes of miR-1307-3p.

### Pull-Down Assay With Biotinylated CircPPP1CB Probe

Bladder cancer cells (1 × 10^7^) were obtained, lysed, and sonicated as indicated. To generate the probe-coated beads, C-1 magnetic beads (Life Technologies) were coincubated with the circPPP1CB probe for 2.5 h at 25°C. Then, the circPPP1CB probe or oligo probe was coincubated with cell lysates at 4°C overnight. RNA was eluted and extracted by wash buffer and used for qRT-PCR. The circPPP1CB probe with biotinylation was synthesized and purchased from RiboBio (Guangzhou, China).

### Luciferase Reporter Assay

First, a luciferase reporter vector (pGL3-Firefly_Luciferase-Renilla_Luciferase) with SMG1 3’-UTR or circPPP1CB was built, and the mutant vectors were constructed by GeneChem (Shanghai, China). A luciferase vector and miR-1307-3p mimic or mimic negative control was cotransfected into cells and incubated for 24 h. A dual-luciferase reporter assay detection kit (Promega, WI, United States) was used to detect firefly and Renilla luciferase activities, which were measured on a Fluoroskan Ascent device (Thermo Fisher Scientific, United States).

### Colony Formation Assay

Cells were counted and seeded into 12-well plates (500 cells per well). After 8 days of incubation, cell colonies were fixed in 4% paraformaldehyde and stained with crystal violet. Only cell colonies containing 50 cells or more were counted and recorded.

### Migration and Invasion Assay

For the migration assay, 200 μl serum-free medium containing 3 × 10^4^ cells was added into the upper chambers of Transwells (Costar, NY, United States) for 24 h. Similarly, for the invasion assay, cells were added to Matrigel (BD, MA, United States)-precoated Transwell upper chambers and incubated for 48 h. A total of 600 μl RPMI 1640 medium with 10% FBS were added to the bottom chambers as an attractant. Migrated or invaded cells were fixed in 4% paraformaldehyde and then stained with crystal violet. After removing the remaining cells on the upper surface of the membrane, the stained cells were visualized and imaged by microscopy (100×) in five randomly chosen fields.

### Cell Scratch Assay

Cells were seeded into 12-well plates with Culture-Insert (ibidi, Germany) according to the manufacturer’s instructions. The next day, Culture-Inserts were removed, and cells were incubated without serum for another 24 h. Images were recorded at 0 and 24 h on a microscope (Nikon, Tokyo, Japan), and the migration rate of the cells was measured using ImageJ software.

### Tube Formation Assay

Human umbilical vein endothelial cells were seeded into a 96-well plate (2 × 10^4^ per well) pre-coated with Matrigel (BD Biosciences, United States). Conditioned media (CM) acquired from different cells was added into the wells with 6 h of incubation. The formation of tubes was observed by a phase contrast microscopy (Nikon, Japan) and quantified by ImageJ in three randomly selected fields.

### Immunofluorescence Staining

For immunofluorescence staining, cells were permeabilized in 1% Triton X-100 and then blocked with 1% goat serum. The fixed cells were incubated with mouse monoclonal anti-vimentin (Cell Signaling Technology, Beverly, NJ, United States) as the primary antibody and Alexa Fluor 488 goat anti-mouse IgG (Thermo Fisher Scientific, United States) as the secondary antibody. Cell nuclei were stained with DAPI. Fluorescence images were captured with a Zeiss Axio Observer A1 (Carl Zeiss, Germany).

### Western Blot Analysis and Antibodies

Briefly, total protein was extracted using RIPA buffer (C1053; APPLYGEN, Beijing, China). After measurement of protein concentrations, equal amounts of proteins (30 μg) were separated by SDS-PAGE, transferred to polyvinylidene fluoride (PVDF) membranes (Millipore, Billerica, MA, United States), and incubated with 5% skim milk for 2 h at room temperature. After washing the membrane with TBST three times, primary antibodies were coincubated with the membrane at 4°C overnight. The next day, the membrane was incubated with secondary antibody (1:5,000) for 2 h at room temperature. Bands were detected by a Bio-Rad CD Touch detection system. The antibodies used in this study were as follows: anti-GAPDH (Catalog number: 5174), anti-fibronectin (Catalog number: 26836), anti-E-cadherin (Catalog number: 3195), anti-N-cadherin (Catalog number: 13116), and anti-vimentin (Catalog number: 5741) were all obtained from Cell Signaling Technology; anti-SMG1 (ab30916) was purchased from Abcam (Cambridge, United Kingdom); and anti-MMP2 (Catalog number: 10373-2-AP) was obtained from Proteintech (Chicago, United States).

### RNA Immunoprecipitation

RNA immunoprecipitation experiments were performed using the Magna RIP RNA-Binding Protein Immunoprecipitation Kit (Millipore, Billerica, MA, United States). HEK-293 cells were transfected with the Ago2 plasmid or vector. Then, 1 × 10^7^ cells were pelleted and resuspended in 100 μl of RIP Lysis buffer combined with a protease inhibitor cocktail and RNase inhibitors. The cell lysates (200 μl) were incubated with 5 μg of antibody against Ago2 (Millipore) or rabbit IgG-coated beads and rotated at 4°C overnight. After treating the lysates with proteinase K buffer, immunoprecipitated RNA was extracted by using the RNeasy MinElute Cleanup Kit (Qiagen) and reverse transcribed using Prime-Script RT Master Mix (TaKaRa). The abundance of circPPP1CB was detected by qRT-PCR.

### Other *in vitro* Experiments

Other *in vitro* experiments, such as CCK-8 assay, H&E staining, and IHC, have been previously described by us (Wang et al., 2020a, 2021).

### Experimental Animal Models

To establish the orthotopic xenograft tumor models, 4-week-old female BALB/c nude mice were obtained and randomly divided into three groups (*n* = 4 for each group). An equal amount of T24 cells (4 × 10^6^) was subcutaneously injected into the nude mice. Four weeks later, tumors were harvested from mice. Tumor volume (mm^3^) = ab^2^/2.

### Statistical Analysis

Statistical analyses were performed using SPSS 20 (Abbott Laboratories, Chicago, IL, United States). All data are presented as mean ± standard deviation (SD). Student’s *t*-test and Chi-square test were used to analyze differences between the two groups. A paired *t*-test was used to analyze expression levels of circPPP1CB and miR-1307-3p in cancer tissues and matched adjacent normal tissues. Pearson’s analysis was used to clarify the relationship between circPPP1CB and miR-1307-3p. A *p*-value < 0.05 indicated meaningful results in this study.

## Data Availability Statement

The datasets presented in this study can be found in online repositories. The names of the repository/repositories and accession number(s) can be found below: https://www.ncbi.nlm.nih.gov/, GSE150142.

## Ethics Statement

The studies involving human participants were reviewed and approved by the Ethics Committee of the First Affiliated Hospital, Zhejiang University School of Medicine. The patients/participants provided their written informed consent to participate in this study. The animal study was reviewed and approved by the Ethics Committee of The First Affiliated Hospital, Zhejiang University School of Medicine.

## Author Contributions

XJ, GD, and FW conceived and designed the study. FW, MF, XZ, YY, and NH performed the experiments. FW, MF, YZ, HW, and SH conducted the statistical analyses. FW wrote the manuscript. XJ and ZH revised the manuscript. FW, MF, YC, XZ, and YY revised the manuscript and conducted the *in vitro* and *in vivo* experiments. All authors contributed to the article and approved the submitted version.

## Conflict of Interest

The authors declare that the research was conducted in the absence of any commercial or financial relationships that could be construed as a potential conflict of interest.

## Publisher’s Note

All claims expressed in this article are solely those of the authors and do not necessarily represent those of their affiliated organizations, or those of the publisher, the editors and the reviewers. Any product that may be evaluated in this article, or claim that may be made by its manufacturer, is not guaranteed or endorsed by the publisher.

## Footnotes

1
https://circinteractome.nia.nih.gov/

2
http://www.circbank.cn

3
http://www.targetscan.org/vert_72/

## Supplementary Material

Supplementary Figure 1 Inhibition of circPPP1CB promoted proliferation, angiogenesis, migration, and invasion of bladder cancer cells. **(A)** CCK-8 assays indicated that knockdown of circPPP1CB promoted cell proliferation rate in bladder cancer cells. **(B)** Colony formation was evaluated by colony formation assay in negative control or circPPPC1B-knockdown cells. **(C)** Tube formation assay was conducted in HUVEC cells with different conditional medium. Scale bars, 400 μm. **(D,E)** Transwell migration and invasion assays and cell scratch assay indicated the biological effects of circPPP1CB knockdown in T24 and EJ cells. Scale bars, 200 and 400 μm, respectively. **(F)** EMT markers (Fibronectin, N-cadherin, E-cadherin, and Vimentin) and MMP2 were detected by western blotting in different T24 and EJ cells. **(G)** Immunofluorescence analysis of Vimentin in circPPP1CB-depletion cells and negative control cells. Scale bars, 200 μm. ^∗^*P* < 0.05, ^∗∗^*P* < 0.01 vs. control group.

Supplementary Figure 2 Depletion of miR-1307-3p suppressed proliferation, angiogenesis, migration and invasion of bladder cancer cells. **(A)** CCK-8 assays showed that knockdown of miR-1307-3p inhibited cell proliferation rate of bladder cancer cells. **(B)** Colony formation assay was used to verify the effects of miR-1307-3p on colony formation ability. **(C)** Different conditional medium from control and miR-1307-3p knockdown cells was collected for tube formation assay. Scale bars, 400 μm. **(D,E)** Transwell migration and invasion assays and wound healing assay indicated that miR-1307-3p served an important role in cell migration and invasion. Scale bars, 200 μm and 400 μm, respectively. **(F)** EMT process and cell adhesion were detected by western blotting in different T24 and EJ cells. **(G)** The expression level of Vimentin was determined by immunofluorescence in miR-1307-3p-depletion cells and control cells. Scale bars, 200 μm. ^∗^*P* < 0.05, ^∗∗^*P* < 0.01 vs. control group.

Supplementary Figure 3 MiR-1307-3p/SMG1 axis regulated biological functions in human bladder cancer. **(A,B)** Cell proliferation and colony formation ability were evaluated in cells under different conditions by CCK-8 assay and colony formation assay, respectively. **(C)** HUVECs were cultured with conditional media acquired from different bladder cancer cells to determine effects of miR-1307-3p and SMG1 on tumor angiogenesis. Scale bars: 400 μm. **(D–F)** The biological effects of miR-1307-3pp and SMG1 were determined by Transwell assay (Scale bars: 200 μm), cell scratch assay (Scale bars: 400 μm) and western blotting. **(G)** Expression levels of Vimentin in cells were detected by Immunofluorescence assay. Scale bars: 200 μm. ^∗^*P* < 0.05, ^∗∗^*P* < 0.01 vs. control group.

Supplementary Figure 4 Detection of PPP1CB protein level in different cells. **(A,B)** Western blotting was performed to determine the alternations of PPP1CB in different cells at protein level.
