# Supplementary figures and images for: Circular RNA CircPPP1CB Suppresses Tumorigenesis by Interacting With the MiR-1307-3p/SMG1 Axis in Human Bladder Cancer
Source: Front Cell Dev Biol. 2021 Sep 14;9:704683. doi: 10.3389/fcell.2021.704683 (PMC8476764; doi:10.3389/fcell.2021.704683)

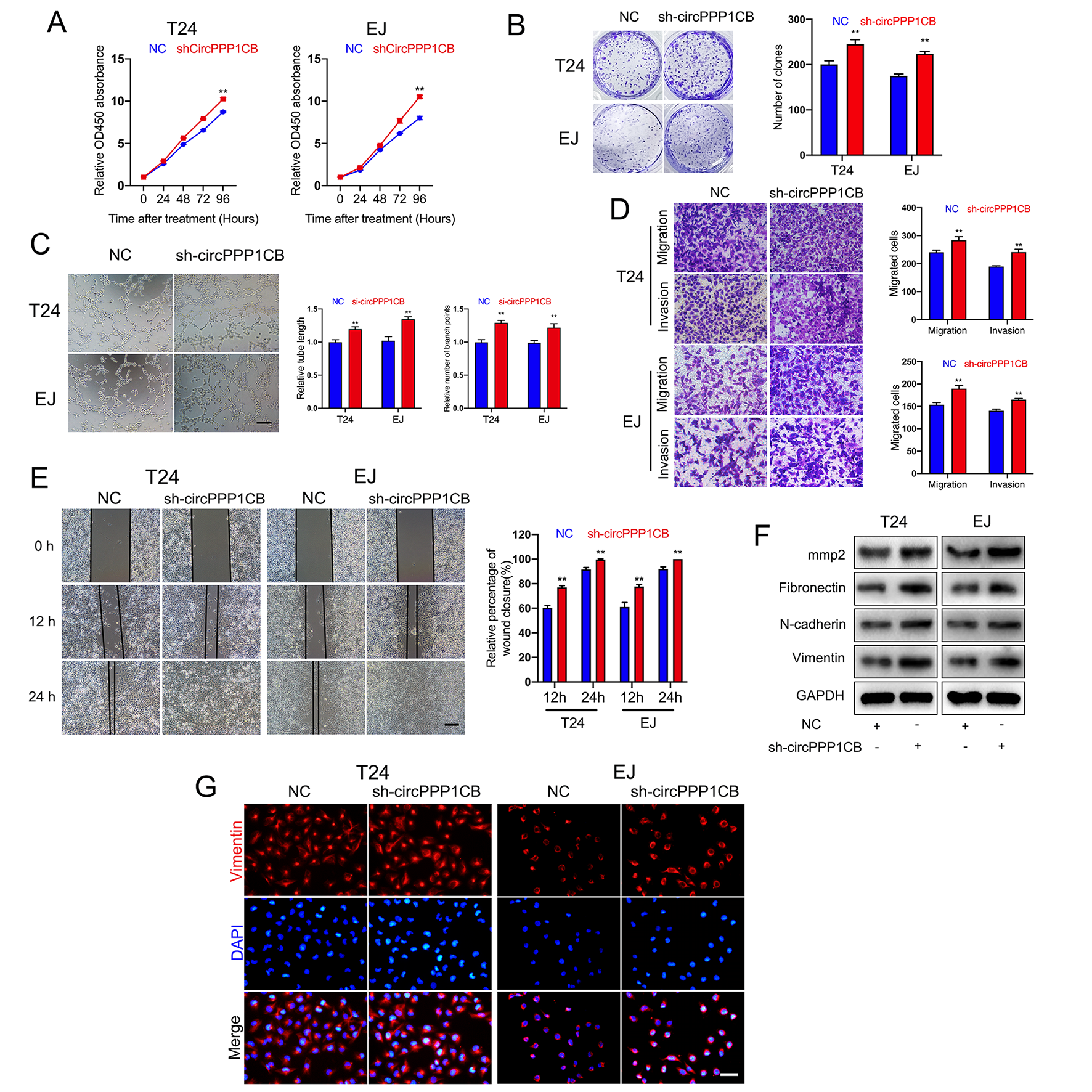

Supplement: Supplementary Figure 1 — Inhibition of circPPP1CB promoted proliferation, angiogenesis, migration, and invasion of bladder cancer cells. (A) CCK-8 assays indicated that knockdown of circPPP1CB promoted cell proliferation rate in bladder cancer cells. (B) Colony formation was evaluated by colony formation assay in negative control or circPPPC1B-knockdown cells. (C) Tube formation assay was conducted in HUVEC cells with different conditional medium. Scale bars, 400 μm. (D,E) Transwell migration and invasion assays and cell scratch assay indicated the biological effects of circPPP1CB knockdown in T24 and EJ cells. Scale bars, 200 and 400 μm, respectively. (F) EMT markers (Fibronectin, N-cadherin, E-cadherin, and Vimentin) and MMP2 were detected by western blotting in different T24 and EJ cells. (G) Immunofluorescence analysis of Vimentin in circPPP1CB-depletion cells and negative control cells. Scale bars, 200 μm. ∗P < 0.05, ∗∗P < 0.01 vs. control group. [file Image_1.TIF]

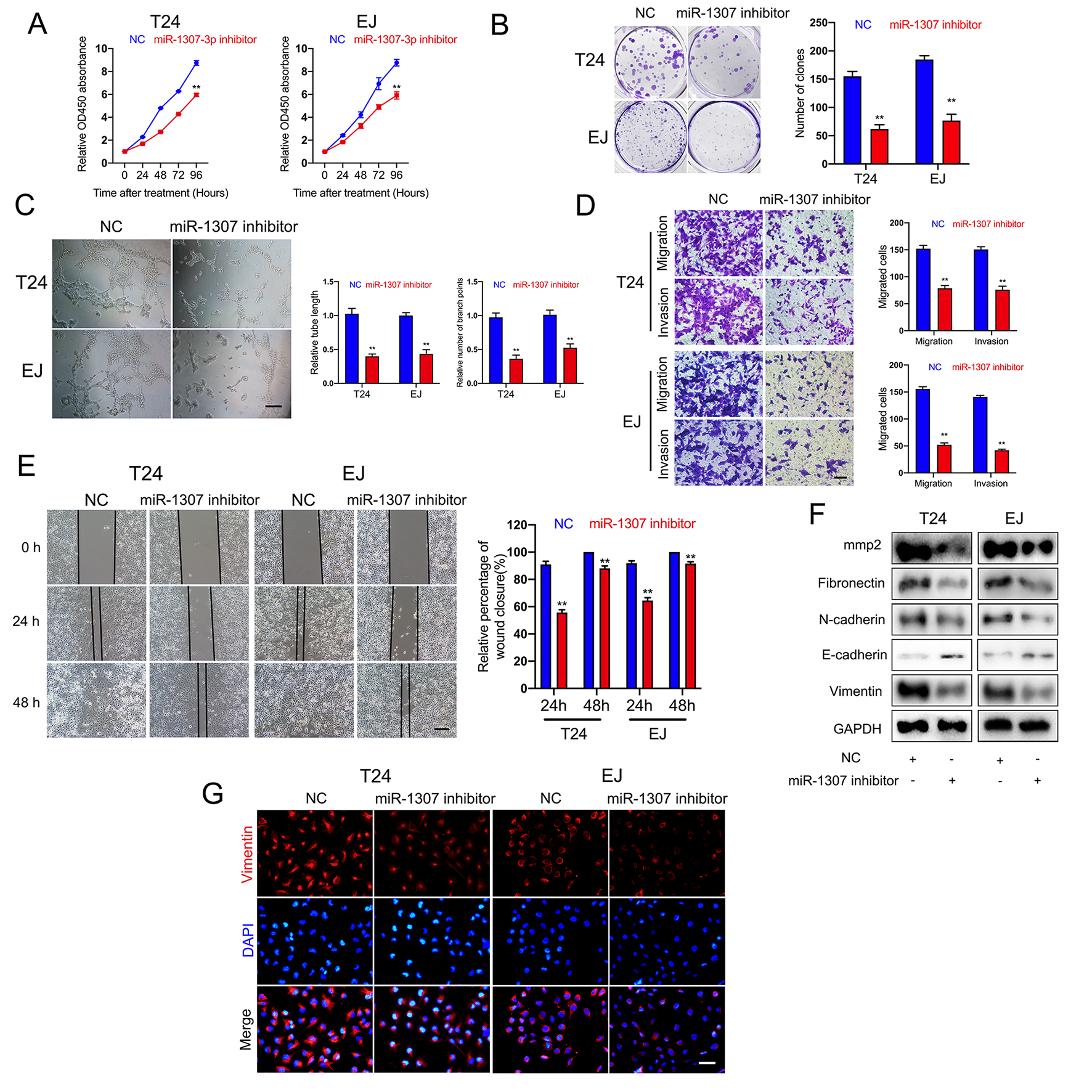

Supplement: Supplementary Figure 2 — Depletion of miR-1307-3p suppressed proliferation, angiogenesis, migration and invasion of bladder cancer cells. (A) CCK-8 assays showed that knockdown of miR-1307-3p inhibited cell proliferation rate of bladder cancer cells. (B) Colony formation assay was used to verify the effects of miR-1307-3p on colony formation ability. (C) Different conditional medium from control and miR-1307-3p knockdown cells was collected for tube formation assay. Scale bars, 400 μm. (D,E) Transwell migration and invasion assays and wound healing assay indicated that miR-1307-3p served an important role in cell migration and invasion. Scale bars, 200 μm and 400 μm, respectively. (F) EMT process and cell adhesion were detected by western blotting in different T24 and EJ cells. (G) The expression level of Vimentin was determined by immunofluorescence in miR-1307-3p-depletion cells and control cells. Scale bars, 200 μm. ∗P < 0.05, ∗∗P < 0.01 vs. control group. [file Image_2.TIF]

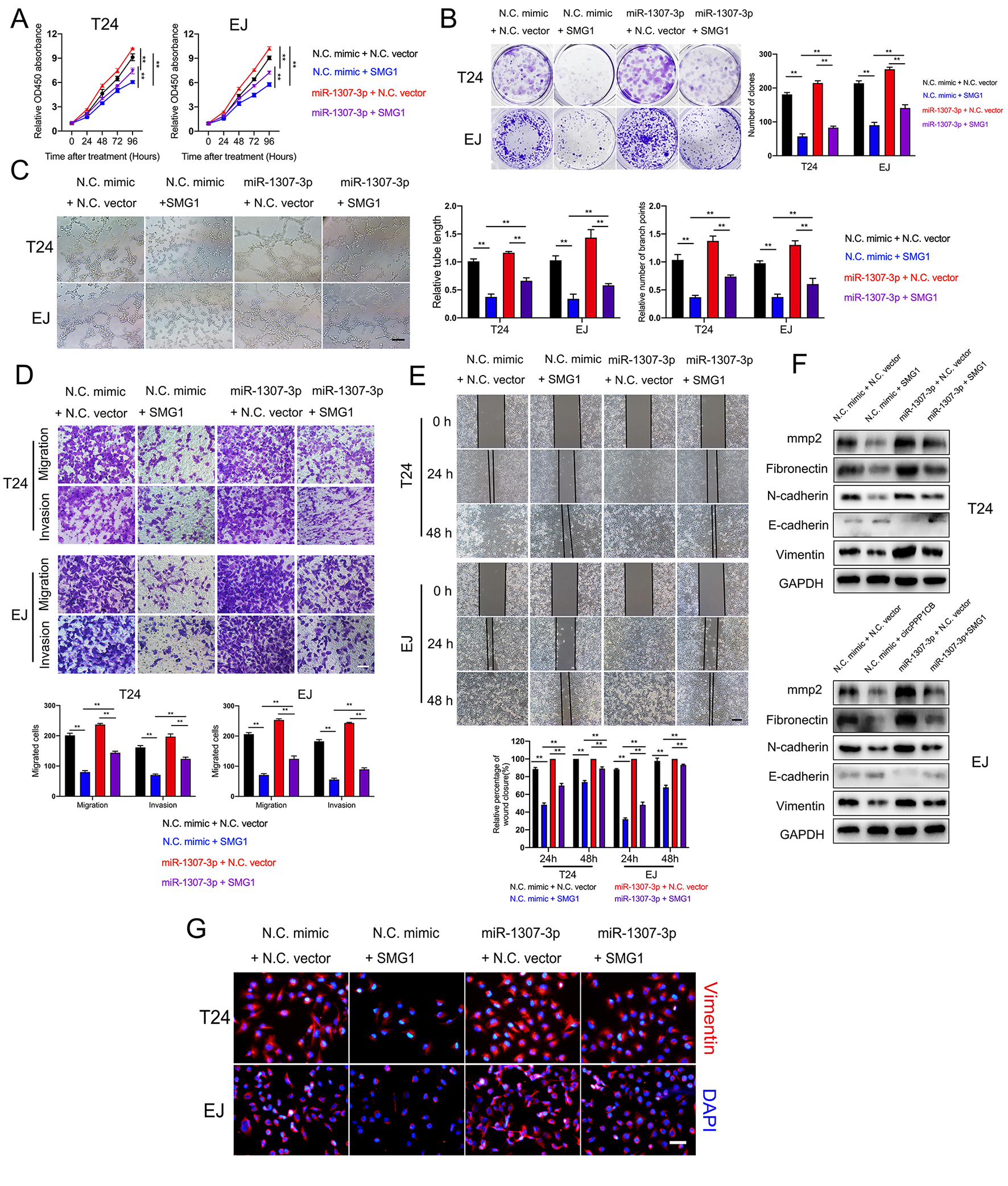

Supplement: Supplementary Figure 3 — MiR-1307-3p/SMG1 axis regulated biological functions in human bladder cancer. (A,B) Cell proliferation and colony formation ability were evaluated in cells under different conditions by CCK-8 assay and colony formation assay, respectively. (C) HUVECs were cultured with conditional media acquired from different bladder cancer cells to determine effects of miR-1307-3p and SMG1 on tumor angiogenesis. Scale bars: 400 μm. (D–F) The biological effects of miR-1307-3pp and SMG1 were determined by Transwell assay (Scale bars: 200 μm), cell scratch assay (Scale bars: 400 μm) and western blotting. (G) Expression levels of Vimentin in cells were detected by Immunofluorescence assay. Scale bars: 200 μm. ∗P < 0.05, ∗∗P < 0.01 vs. control group. [file Image_3.TIF]

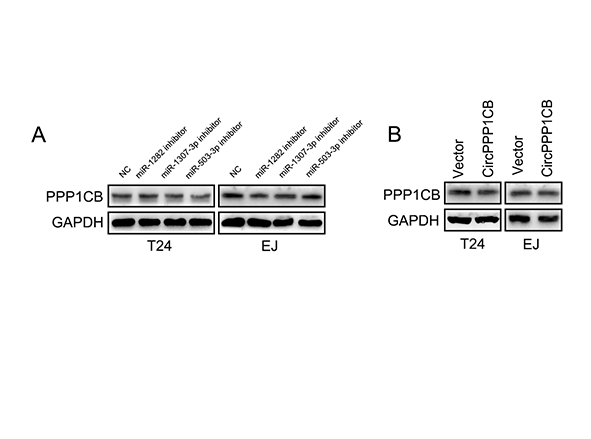

Supplement: Supplementary Figure 4 — Detection of PPP1CB protein level in different cells. (A,B) Western blotting was performed to determine the alternations of PPP1CB in different cells at protein level. [file Image_4.TIF]
