# Supplementary material for: Circular RNA CircPPP1CB Suppresses Tumorigenesis by Interacting With the MiR-1307-3p/SMG1 Axis in Human Bladder Cancer
Source: Front Cell Dev Biol. 2021 Sep 14;9:704683. doi: 10.3389/fcell.2021.704683 (PMC8476764; doi:10.3389/fcell.2021.704683)
Supplement: Supplementary file 5 [file Table_1.docx]

**Table S1. The sequences of primers and oligonucleotides used in this study**

| **Primers for RT-qPCR** | |
| --- | --- |
| GAPDH-cp F | GAACGGGAAGCTCACTGG |
| GAPDH-cp R | GCCTGCTTCACCACCTTCT |
| GAPDH-dp F | CCTCTGACTTCAACAGCGACAC |
| GAPDH-dp R | CCATCACGCCACAGTTTCC |
| circ_0119704 F | TCCACCTCGAACAGCTAATCC |
| circ_0119704 R | TGCTTCAGTCATCTGCACAATC |
| circ_0119704-cp F | GAATATGGAGGTTTCCCACCAG |
| circ_0119704-cp F | ATCATAGAATCCATAAATGCGATTG |
| **Primers for Real time-qPCR** | |
| circ-PPP1CB F | TCCACCTCGAACAGCTAATCC |
| circ-PPP1CB R | TCTTGTCGGCACAGACTCTCC |
| Linear PPP1CB F | GCTGCTGGAGGTACGAGGATGT |
| Linear PPP1CB R | AGTTGGCTTCTGGTGGGAAACC |
| GAPDH F | AGAAGGCTGGGGCTCATTTG |
| GAPDH R | GCAGGAGGCATTGCTGATGAT |
| ISG20L2 F | GCGGAGGCTCTTAGAACGGAGA |
| ISG20L2 R | GCGGGTTGGGTGGCTATTGAT |
| PIAS4 F | TCTCCGACCTTCAGATGCTCCT |
| PIAS4 R | CACCTGTCTTGGCGTCAATGC |
| CIC F | CCGAGGATGCTGAGGTCTCTAAGAT |
| CIC R | CGCCACTGCTTGCCGTTGAA |
| PRPF4 F | GCGGCTGATGGCTCTGTGAA |
| PRPF4 R | TGAACGGTCATAGCAGGTGGTG |
| ORC6 F | GCAGTCATGTGCCTGGACCTTG |
| ORC6 R | GGGCTTCAACCACTATCTTCTTTCTCTT |
| DNAJC22 F | AGTGTGCGGCTCTATCGTCTGG |
| DNAJC22 R | AAGTGCCTCTGTGCCTCCTCTG |
| WDR92 F | AGCCTCAGATCATTGTCCACATTCA |
| WDR92 R | TGCCACGGTCCAACAGTCTCT |
| SLC35E1 F | AAGGTCTTGCGAGATTCACGGATC |
| SLC35E1 R | CGGCTGTATTGGAAGTGGTCTGTTA |
| YIPF5 F | GCAGCCACAACAGCCATACAC |
| YIPF5 R | AAGGACACTTGCCACACAACCA |
| RAB27A F | CGACAGCGTTCTTCAGAGATGC |
| RAB27A R | CTGCGAGTGCTATGGCTTCCT |
| ZNF333 F | AGGAGTGGGCATTGCTGGACA |
| ZNF333 R | CAACACCTGTGGCACGGAGAAT |
| DIP2A F | AGCCGTCTACACTCTCACCTATGG |
| DIP2A R | ACCTCTCCTGTCTGTGCCTTGG |
| LDLR F | GCTCCATCGCCTACCTCTTCTTCA |
| LDLR R | CCTCTCACACCAGTTCACTCCTCTT |
| PHLDA3 F | CTCAGCTTCGCCCGCATCAA |
| PHLDA3 R | GCCTGCTGGTTCTTGAACTTGAC |
| TSPAN14 F | TGCCAGCTACAGCCGAGAGAAG |
| TSPAN14 R | CGATGAAGACGCCAGCCACAAT |
| ERC1 F | GGCTGACACCACCAACACTGAC |
| ERC1 R | ACACTCCTCCTTCTTCTGCTCCAA |
| ZNF695 F | TCATCTGTCTGGAGGCAAGGAA |
| ZNF695 R | AGCAAGCACATCTTCACATGGA |
| ERGIC1 F | ACGCTACAGGTCCAGAACATCCA |
| ERGIC1 R | CTGCTTGCCACTCTTGTCCTCATAA |
| SYNRG F | CAGAGAACTTGAACAGACAGCAGAGAA |
| SYNRG R | TGGTGTGAGTGTGGCGAGTGA |
| ZNF652 F | CAGTTCATGTGCCAGTGGTGTG |
| ZNF652 R | GTGTGAGTTCTGCGGTGTCTCT |
| SMG1 F | CTGCTGCGGTTGCTCGTGAA |
| SMG1 R | CGTAAGGTGTTGTTGTTCTGGACTCT |
| SWSAP1 F | CTGCTGCTCGGTACACCAGGAT |
| SWSAP1 R | AGGCAATGAGGTAGGCGGCTTC |
| FOXK1 F | CCGCCTCCATCGTAACCTCACA |
| FOXK1 R | CCGAGTTGGCAGATGTGGTGAC |
| ST3GAL2 F | TGGACGGGCACAACTTCATCAT |
| ST3GAL2 R | GGCAGGTTCTTGGCACTCTCAG |
| APOPT1 F | TCTCAAGATTCTGCCCTCCAAG |
| APOPT1 R | TTCCTTGTAGAAGTCCGCCATT |
| Hsa-miR-1282 | TCGTTTGCCTTTTTCTGCTT |
| Hsa-miR-1307-3p | ACTCGGCGTGGCGTCGGTCGTG |
| Hsa-miR-338-3p | TCCAGCATCAGTGATTTTGTTG |
| Hsa-miR-503-3p | GGGGTATTGTTTCCGCTGCCAGG |
| Hsa-miR-548p | TAGCAAAAACTGCAGTTACTTT |
| miRNA reverse | All-in-One miRNA qRT-PCR detection kit, GeneCopoeia, USA |
| **siRNAs sequence** | |
| Si SMG1 | TACATTGTTGATGATAGATTAAC |
| Si YIPF5 | CAGTTATAGTTACGAGAAAGATA |
| Si PRPF4 | AGGATGTACTTCGTGTATTAAAG |
| Circ_0119704 probe | CCGAAGAAAAGGTGAGAGAAC |
| Hsa-miR-1307-3p probe | CACGACCGACGCCACGCCGAGT |
| Hsa-miR-1307-3p mimic | ACUCGGCGUGGCGUCGGUCGUG |
| Hsa-miR-1282 inhibitor | AAGCAGAAAAAGGCAAACGA |
| Hsa-miR-1307-3p inhibitor | CACGACCGACGCCACGCCGAGT |
| Hsa-miR-503-3p inhibitor | CCTGGCAGCGGAAACAATACCCC |
